# Trends in black fly density, parity and infection rates from riverside to villages of the Bafia Health District in Cameroon: implication for onchocerciasis vector control

**DOI:** 10.1186/s13071-023-05832-y

**Published:** 2023-08-04

**Authors:** André Domche, Hugues C. Nana-Djeunga, Philippe B. Nwane, Guy R. Njitchouang, Dirane C. Dzune-Fossouo, Betrand Nono Fesuh, Flobert Njiokou, Joseph Kamgno

**Affiliations:** 1 Higher Institute for Scientific and Medical Research (ISM), Yaoundé, Cameroon; 2 Parasitology and Ecology Laboratory, Department of Animal Biology and Physiology, Faculty of Science, University of Yaoundé 1, Yaoundé, Cameroon; 3 National Advanced School of Engineering, University of Yaoundé I, Yaoundé, Cameroon; 4 Faculty of Medicine and Biomedical Sciences, University of Yaoundé 1, Yaoundé, Cameroon

**Keywords:** Onchocerciasis, Blackfly, Vector control, Esperanza window trap

## Abstract

**Background:**

Reducing contact between humans and black flies can lead to interruption of onchocerciasis transmission. The Esperanza Window Trap (EWT) has been shown to be an effective tool for reducing black fly densities. Several shape-based improvements to this trapping system have been made to optimise its effectiveness, but optimisation of this trapping system has been based most often on the shape of the trap, collection in areas of high black fly density and the addition of attractants, without considering transmission potentials and parity rates. This study aims to investigate the differences in biting rates and transmission potential between three catch points along a transect to guide the choice of EWT placement.

**Methods:**

Monthly black fly collection was carried out over a 1-year study period using the human landing method at three catch points along a transect from the riverside toward the centre of two first-line villages (Biatsota and Bayomen), in the Mbam valley in Cameroon. All female black flies caught were counted and dissected, and entomological indicators were computed and compared between the catch points and villages.

**Results:**

A total of 80,732 black flies were caught, of which 57,517 were dissected; of the latter, 2743 (4.8%) were parous and 44 (1.6%) were infective. Regarding the distance to the river, a vector density gradient was observed, with the highest annual biting rates being recorded at the riverside. The highest annual transmission potentials were also recorded at the riverside (165 vs 255 infective larvae/man/year in Bayomen and Biatsota, respectively). Overall, the highest parity rates were recorded at the riverside in Biatsota (5.1%) where various human activities are frequent and at the centre of Bayomen village (6.3%).

**Conclusion:**

The results of this study reveal that entomological parameters were the highest at the riverside catch sites and indicate that riverside locations should be prioritised for EWTs or other trapping systems to achieve optimal performance in onchocerciasis control.

**Graphical abstract:**

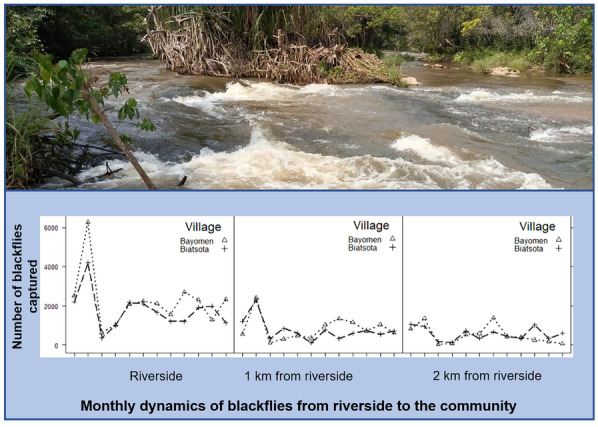

## Background

Human onchocerciasis, commonly known as river blindness, is a parasitic disease caused by the filarial nematode *Onchocerca volvulus,* which is transmitted by black flies during their blood meals. This parasitic disease is the second leading infectious cause of blindness worldwide and remains a major public health concern in many sub-Saharan African countries [1]. River blindness is currently endemic in 31 countries in sub-Saharan Africa, Yemen and Latin America [2]. The geographical distribution of onchocerciasis is generally aligned to the geographical distribution of its black fly vectors. Inhabitants of first-line villages (i.e. those that are closest to the river where black fly breeding sites are located) are generally the most affected by the disease. Because of the nuisance caused by black fly bites, the majority of villages and their inhabitants are generally found > 1 km distant from the river where breeding sites are located, with the consequence that the most fertile lands are unoccupied [3].

Nowadays, onchocerciasis control relies essentially on mass distribution of the antiparasitic drug ivermectin in eligible areas through the strategy known as Community-Directed Treatment with Ivermectin (CDTI) [4]. However, in areas not eligible for CDTI, and in areas where onchocerciasis transmission persists despite several years of annual mass treatment, alternative strategies are recommended, including vector control [5], to accelerate transmission interruption. Vector control can lead to the reduction of contact between man and black fly to a very low level, and therefore contribute to transmission interruption of onchocerciasis or its reduction to levels at which serious clinical signs of the disease are unlikely to occur [6]. Vector control can target: (i) immature aquatic stages using larvicides as was the case for the Onchocerciasis Control Program in West African (OCP) [7] or the innovative and sustainable “slash and clear” approach recently developed in Uganda [8]; and/or (ii) adult black flies, through aerial spraying of insecticides or through trapping [6, 9]. Several studies have demonstrated the ability of the Esperanza Window Trap (EWT) to reduce black fly densities [9, 10]. However, when developing modifications for optimisation of this trapping system, the main parameters usually taken into account have been the shape of the trap and the addition of attractants [9, 10], even though transmission potential is an important parameter in the epidemiology of onchocerciasis. Therefore, the effectiveness of EWT could be optimised by placing them in areas where the biting rates and transmission potentials are the highest.

The aim of this study was therefore to investigate the differences in biting rates and transmission potentials between three catch points along a transect to guide the choice of EWT placement for an optimal reduction of black fly population densities and onchocerciasis transmission in two first-line villages of the Bafia Health District, Centre Region, Cameroon.

## Methods

### Study area and population

**Fig. 1:**
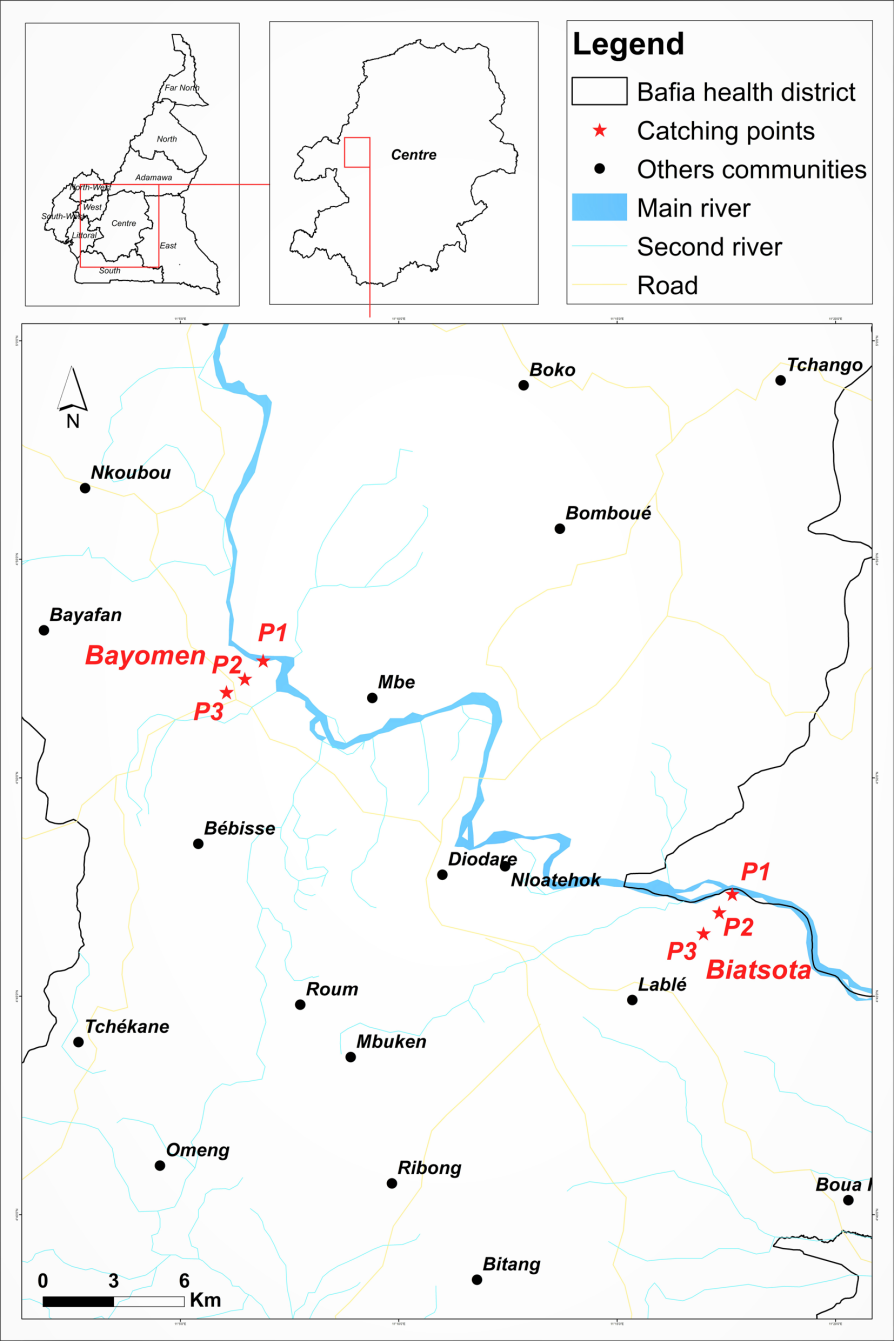
Study area showing the catch points. P1, Catch points in both villages located at the riverside; P2, catch points in both villages located at about 1 km from the riverside; P3, catch points in both villages located at about 2 km from the riverside

The study was conducted in two first-line villages situated approximately 22 km apart: Bayomen (4.86499N, 11.10804E) and Biatsota (4.77640N, 11.28884E), of the Bafia Health District, located in the Mbam-and-Inoubou Division, Centre Region, Cameroon (Fig. 1). It is a forest-savannah transition zone with many fast-flowing rivers, such as the Sanaga River and its main tributary, the Mbam river, both of which exhibit on their courses several falls and rapids suitable for the breeding of black flies. Onchocerciasis is known to be endemic in the Bafia Health District, with reported microfilaridermia prevalence varying from 24.4% to 57.0% despite > 15 years of CDTI [11]. In this area, onchocerciasis has been also associated with a high prevalence of blindness, epilepsy and excess mortality [12, 13]. Entomological studies across the study areas showed high biting rates of the black fly* Simulium damnosum* sensu lato (*S. damonsum* s.l.), mostly during the rainy season [14, 15]. The control of onchocerciasis in the Bafia Health District is based solely on mass administration of ivermectin. In 2005, the Yaoundé Initiative Foundation initiated vector control in the Sanaga Valley, which resulted in the reduction of black fly adult populations for several weeks following larvicide application; unfortunately, a rebound in population densities of the vector was observed as soon as the intervention was halted [14].

In 2017, the population of the Bafia Health District was estimated at about 161,400 inhabitants, with 388 residents in Biatsota and 481 in Bayomen [16]. Overall, the main human activities are agriculture (mainly cocoa), fishing and sand mining along the Mbam river. In Biatsota, a number of various human activities are carried out throughout the day at the riverbank, including crossing the Mbam river by ferry (mainly traders and transporters) and sand mining close to the ferry site. In contrast in Bayomen, almost no human activities occur at the riverbank, apart from those on poorly tended cocoa fields.

### Study design and procedures

A longitudinal entomological study was conducted from October 2019 to September 2020. Black flies were collected, then dissected to assess parity and infectivity. Entomological indices were calculated and compared between the different catch points.

#### Collection of female black flies

Female black flies were caught using the human landing collection method [17]. *Simulium damnosum* s.l. was distinguished from other black fly species based on different morphological features, including the colour of the tufts of setae at the base of the wings, the colour of the antennae, procoxa, prosternum and mesonotum and the shape of the anterior basitarsus [18]. Fly collection was performed on three consecutive days every month for > 1 year. Three catch points (referred to as P1, P2 and P3; Fig. 1) were chosen along a transect, from the riverbank toward the centre of each village (Fig. 1). Catch points 1 (P1) in both villages were located at the riverside, catch points 2 (P2) were located at about 1 km from the riverside and catch points 3 (P3) were located at about 2 km from the riverside. Collections were made at the same time points in both villages and in each of the catch points using mouth aspirators, as previously described [15]. At each catch point, black flies were collected by a team of two volunteers working alternately from 7 AM to 12 PM and from 12 PM to 5 PM. To limit biases related to aptitude and individual attractiveness of fly collectors, the allocation of the fly collectors to catch points was organised on a monthly rotation basis.

#### Dissection of black flies for parity and* O. volvulus* infection

Black flies were dissected individually in a drop of saline under a binocular stereomicroscope by experienced laboratory technicians according to procedure previously described by Wanson [19] and Philippon [20]. The abdomen of each black fly was carefully dilacerated using mounted needles for the examination of the ovaries and Malpighian tubes to differentiate nulliparous flies (females that have never laid eggs) from parous flies (females that have completed ≥ 1 gonotrophic cycles). Nulliparous flies were not processed further and preserved in ethanol for future analyses. Tissues of each body part (head, thorax, abdomen) of parous flies were dissected separately under a binocular stereomicroscope for the presence of larvae morphologically similar to those of*O. volvulus*. The number, stage of development and location of these larvae were recorded. Black flies were considered to be infected when they harboured first-, second- and third-stage larvae (L1, L2 or L3), regardless of their location, and to be infectious when infective L3 were found in the head. All L3 found in the head of each infected fly were harvested and preserved, with the black fly’s mouthparts, between a slide and coverslip, as previously described [21], for further molecular analyses.

#### Black fly infection rates by pool screening

Once in the laboratory, pool screening was used to confirm the *Onchocerca* to species level and to provide a robust estimate of black fly infection rates. The preserved black flies were grouped in pools of 100 specimens in Petri dishes, then dissected under a binocular stereomicroscope to separate the heads from the thorax and abdomen. To prevent contamination, the Petri dishes were changed after each use, and the dissecting instruments (mounted needles) carefully cleaned after the dissection of each pool of blackflies. Pools of 100 heads and 100 bodies (abdomen and thorax) from the same flies were then stored separately in 2-ml microcentrifuge tubes containing absolute ethanol and labelled with the name of the village, site and month of collection. DNA was extracted from the head and abdomen-thorax pools, respectively, using the QIAamp DNA Tissue Kit (QIAGEN Inc., Valencia, CA, USA) following the manufacturer’s instructions. Real-time PCR amplification of *O. volvulus* larvae was performed as previously described [15], in duplicate, in a total reaction volume of 10 μl containing 1 μl template DNA or molecular biology-grade water as a negative control, 0.3 µl of each OvOoND5 (10 µmol/;) forward (5′-GCT ATT GGT AGG GGT TTG CAT-3′) and reverse (5′-CCA CGA TAA TCC TGT TGA CCA-3′) primer, 0.05 µl of dNTPs (10 mM), 0.05 µl Taq DNA polymerase (5 units/µl), 0.1 µl of hybridisation probe (5 µM; Fam-TAA GAG GTT ATT GTT TAT GCA GAT GG-BHQ1), 1 µl of PCR buffer (10×), 1.2 µl of MgCl_2_ (4.5 mM) and 6 µl molecular biology-grade water. Amplification was carried out using the StepOnePlus thermal cycler (Applied Biosystems, Thermo Fisher Scientific, Waltham, MA, USA) with the following cyclin conditions: an initial denaturation (Taq polymerase activation) at 95 °C for 10 min, followed by 45 cycles of denaturation at 95 °C for 10 s and hybridisation at 61 °C for 30 s, with fluorescence acquisition on the FAM channels at the end of the reaction. A positive pool of thorax-abdomen was interpreted as being infected with microfilariae or developing *O. volvulus* larvae, whereas a positive pool of heads was interpreted as containing infective L3 parasites.

#### Computation of entomological indices for onchocerciasis transmission

Entomological indices (biting rates, parity rates (PRs), infection and infective rates, transmission potentials) were computed as previously described [17]. Briefly, monthly biting rate (MBR) was defined as the total number of black flies captured divided by the number of days of capture during a month, multiplied by 30. The annual biting rate (ABR) was calculated as the arithmetic sum of the MBRs for the 12 months of black fly collection. The PR was defined as the proportion of parous flies among the total number of flies dissected. Monthly transmission potential (MTP) was calculated as MBR of the month multiplied by the total number of L3 found in black flies’ heads for the targeted month divided by the total number of flies dissected. The annual transmission potential (ATP) was calculated as the arithmetic sum of the MTPs of the 12 months of black fly collection and processing.

### Statistical analyses

All data collected were recorded into a purpose-built Microsoft Excel (Microsoft Corp., Redmond, WA, USA) database and subsequently exported to R software (version 4.1.2) for statistical analyses R Foundation for Statistical Computing, Vienna, Austria). A negative binomial regression model was used to account for variations in the number of bites (dependent variable recorded as counts) between catch sites, villages and date of collection (independent variables). The non-parametric Kruskal–Wallis test was used to compare MTPs within different catch points in each of the two villages and between catch points located at similar distances from the river, and the Chi-square (*χ*^2^) test with Bonferroni correction was used to compare PRs within different catch points in each of the two villages and between catch points located at similar distances from the river. For all statistical analyses, the threshold for significance was set at 5%. The results of pool screening were expressed as infection (proportion of infected pools of thorax-abdomen) and infectivity (proportion of infected pools of heads) rates with 95% confidence intervals (CIs) at each catch point computed using the Wilson method.

## Results

### Black fly relative abundance and biting rates

**Fig. 2:**
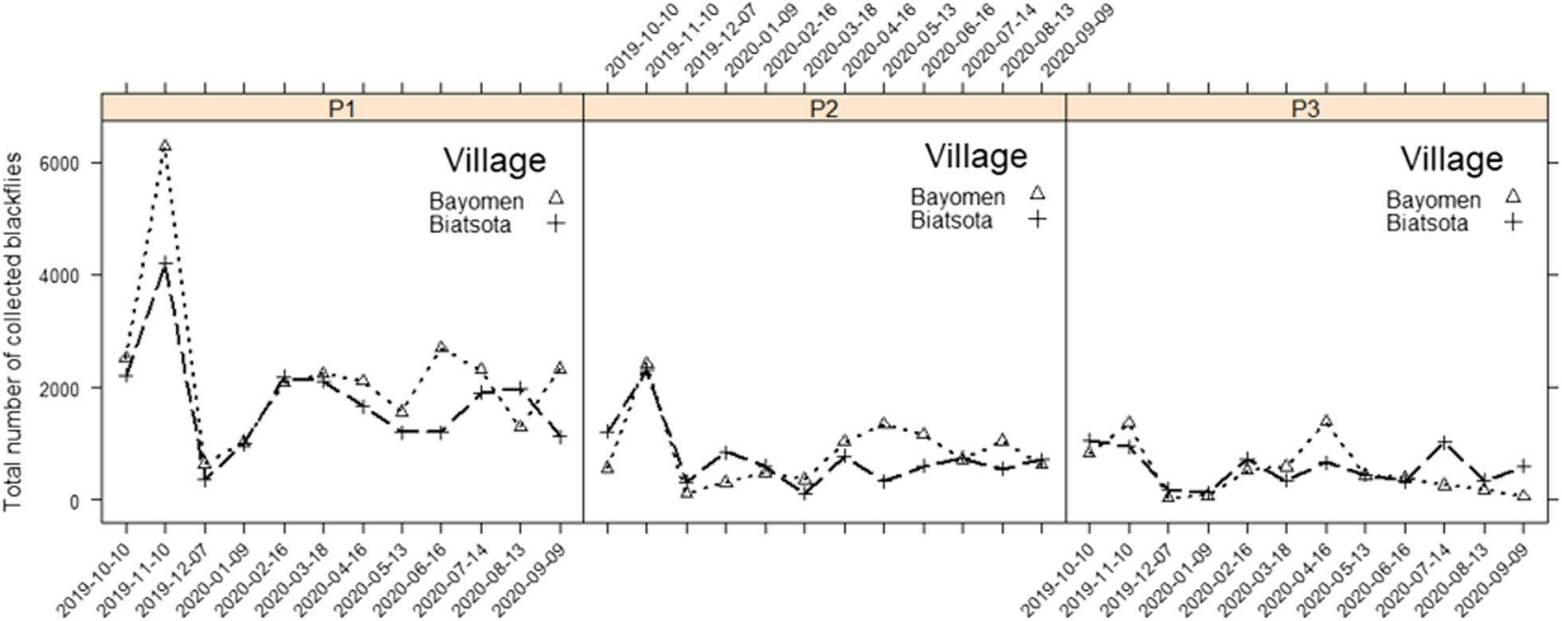
Monthly dynamics of number of blackflies collected in each catch point in the study villages. For explanation of catch points P1, P2, P3, see Fig. 1 caption

The results obtained in each village are summarised in Table 1. From October 2019 to September 2020, 80,732 female black flies (*S. damnosum*) were collected, of which 43,490 (53.9%) were caught in Bayomen and 37,242 in Biatsota. In terms of catch sites, 48,297 female black flies were collected at P1 (27,103 in Bayomen and 21,194 in Biatsota), 19,419 at P2 (10,226 in Bayomen and 9193 in Biatsota) and 13,016 at P3 (6161 in Bayomen and 6855 in Biatsota). A decreasing gradient of biting rates was observed as a function of increasing distance from the Mbam River (P1 biting rates ˃ P2 biting rates ˃ P3 biting rates). Globally, the highest biting rates were observed in November in each catch site (Fig. 2). The highest ABRs were recorded at the riverside (catch point 1) in both villages (274,200 and 214,809 bites/human/year in Bayomen and Biatsota, respectively). ABRs at the riverside site of both villages were more than twofold higher than those at catch point 2 and more than threefold higher than those at the centre of the villages (catch point 3) (Fig. 2). The ordinary negative binomial regression model showed that monthly catches were significantly higher at catch points 1 than at the other catch points in both Bayomen and Biatsota villages (*P* < 0.0001) (Table 2). Although monthly catches were generally higher in Bayomen than in Biatsota, there was no significant difference between these at the points located at similar distances from the riverside in the two villages (*P* > 0.547).

**Table 1:** Summary of black fly total catch and dissection in the three catch points of the study villages

Villages	Catch points^a^	No. of female black flies collected	Annual biting rate	No. of black flies dissected	No. of parous female black flies (%)	No. of black flies with *Onchocerca volvulus* L1–L3	No. with L3 (%)	No. of L3 (total)	No. of L3 in black flies (head)	Annual transmission potential
Bayomen	P1	27,103	274,200	16,137	711 (4.4)	21	10 (0.06)	12	12	165
	P2	10,226	103,586	8350	373 (4.5)	9	3 (0.04)	3	2	44
	P3	6161	57,892	5522	351 (6.36)	16	7 (0.13)	7	7	80
Biatsota	P1	21,194	214,809	14,461	737 (5.1)	28	12 (0.08)	15	15	255
	P2	9193	93,114	6895	323 (4.7)	17	6 (0.08)	6	6	62
	P3	6855	69,497	6152	248 (4)	17	6 (0.09)	6	6	60

*L1, L2, L3* First-, second- and third-stage larvae, respectively

^a^For both villages, catch points 1 (P1) were located at the riverside, catch points 2 (P2) were located at about 1 km from the riverside and catch points 3 (P3) were located at about 2 km from the riverside

**Table 2:** Negative binomial regression model of collected blackflies considering collection date, villages and catch points

Parameter	Estimate	Standard error	*Z*-value	*P*-value
Intercept	7.8844	0.19793	39.833	< 2e-16
*Date*	− 0.0447	0.02209	− 2.022	0.0432
*Village (Reference = Bayomen)*				
Biatsota	− 0.0833	0.15451	− 0.539	0.5897
*Catch site (Reference = P1)*				
P2	− 0.9076	0.18916	− 4.798	1.60e-06
P3	− 1.3130	0.18923	− 6.939	3.95e-12

**Fig. 3:**
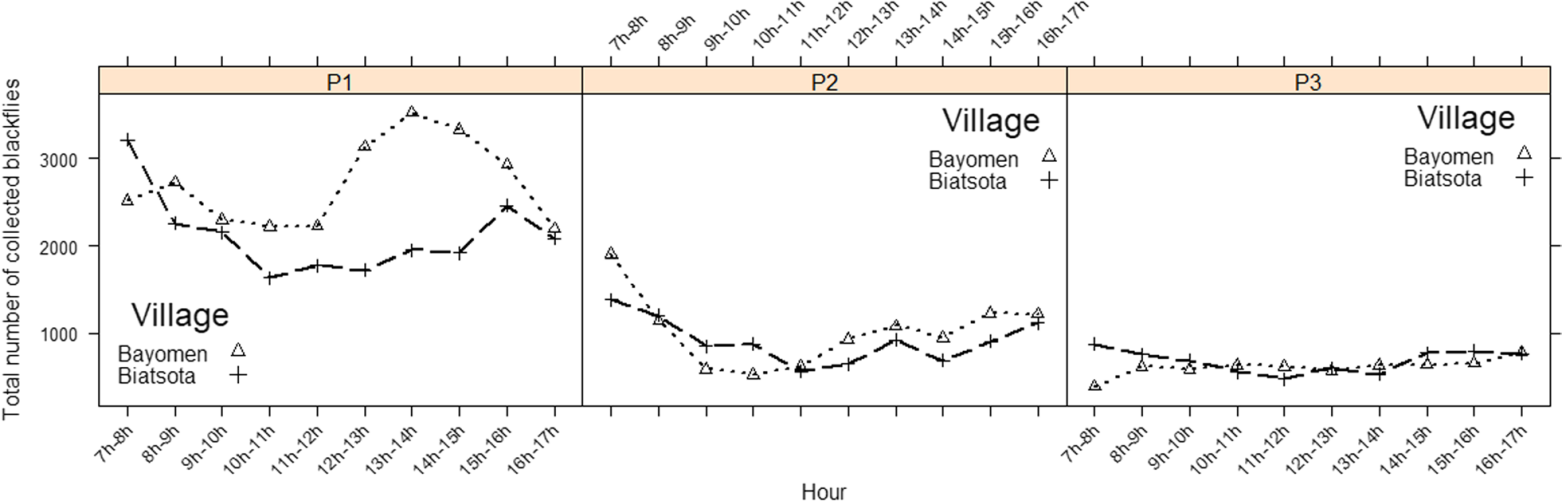
Diurnal biting activity over the 12 months of collection at the three catch points in both villages. For explanation of catch points P1, P2, P3, see Fig. 1 caption

The biting activity of blackflies was quite dynamic during the day, with the highest nuisance being described between 7 AM and 10 AM (with maximal and minimal cumulative biting rates of 3210 and 2158 bites/human/day, respectively, in catch points 1; 1908 and 585 bites/human/day, respectively, in catch points 2; and 875 and 396 bites/human/day, respectively, in catch points 3) and between 1 PM and 5 PM (with a maximal and minimal cumulative biting rates of 3528 and 1954 bites/human/day, respectively, in catch points 1; 1234 and 694 bites/human/day, respectively, in catch points 2; and 785 and 537 bites/human/day, respectively, in catch points 3) (Fig. 3). A similar trend of cumulative black fly diurnal biting activity over the 12 months of collection was globally observed at the three catch points in both villages.

### Parity rates

Of the 80,732 blackflies collected, 57,517 (71.2%) were dissected for parity, including 30,009 (69.0%) caught in Bayomen and 27,508 (73.9%) caught in Biatsota. Globally, PRs at the different catch points ranged between 4.4% and 6.4% in Bayomen and between 4.0% and 5.1% in Biatsota (Table 1). The highest PR was recorded at the riverside in Biatsota (5.1%, 95% CI 4.7–5.4%) and at the centre of the village in Bayomen (6.4%, 95% CI 5.7–7%). The PRs were significantly different between the three catch points, both in Bayomen (*χ*^2^ = 36.89,* df* = 2, *P* < 0.0001) and in Biatsota (*χ*^2^ = 10.91,* df* = 2, *P* = 0.0042). The Bonferroni correction showed significant differences between P1 and P3 and between P2 and P3 in Bayomen (*P* < 0.0001), whereas the only significant difference in Biatsota was observed between P1 and P3 (*P* = 0.0012). In addition, PRs at the catch points located at similar distances from the riverside in the two villages were significantly higher in Biastota than in Bayomen for catch point 1 (*χ*^2^ = 8.06,* df* = 1, *P* = 0.005), but significantly higher in Bayomen than in Biatsota for catch point 3 (*χ*^2^ = 32.320,* df* = 1, *P* < 0.0001).

### Blackfly infection and infectivity rates assessed by pool screening

A total of 140 (77 in Bayomen and 63 in Biatsota) pools of heads and the abdomen-thorax were prepared and analysed using the real-time PCR technique. Table 3 shows the distribution of pools and infection rates according to the catch points in each village. A total of 133/140 (95.0%, 95% CI 90–97.5) abdomen-thorax pools (94.8%, 95% CI 87.3–97.9) in Bayomen and 95.2% (95% CI 86.9–98.3) in Biatsota) were positive to *O. volvulus*; the infectivity rate (proportion of pools of heads positive to *O. volvulus*) was 49.4% (95% CI 38.5–60.3) in Bayomen and 31.7% (95% CI 21.6–44.0) in Biatsota.

**Table 3:** Distribution of pools according to the catch points in each village and *O. volvulus* infection and infectivity rates

Village	Catch point^a^	Total number of pools	*O. volvulus*-positive head pools (%)	*O. volvulus*-positive abdomen-thorax pools (%)
Bayomen	P1	61	34 (55.7)	60 (98.4)
	P2	14	2 (14.3)	11 (78.6)
	P3	02	2 (100)	2 (100)
Biatsota	P1	39	12 (30.8)	38 (97.4)
	P2	18	7 (38.9)	17 (94.4)
	P3	06	1 (16.7)	5 (83.3)

^a^For both villages, catch points 1 (P1) were located at the riverside, catch points 2 (P2) were located at about 1 km from the riverside and catch points 3 (P3) were located at about 2 km from the riverside

### Transmission potentials

**Fig. 4:**
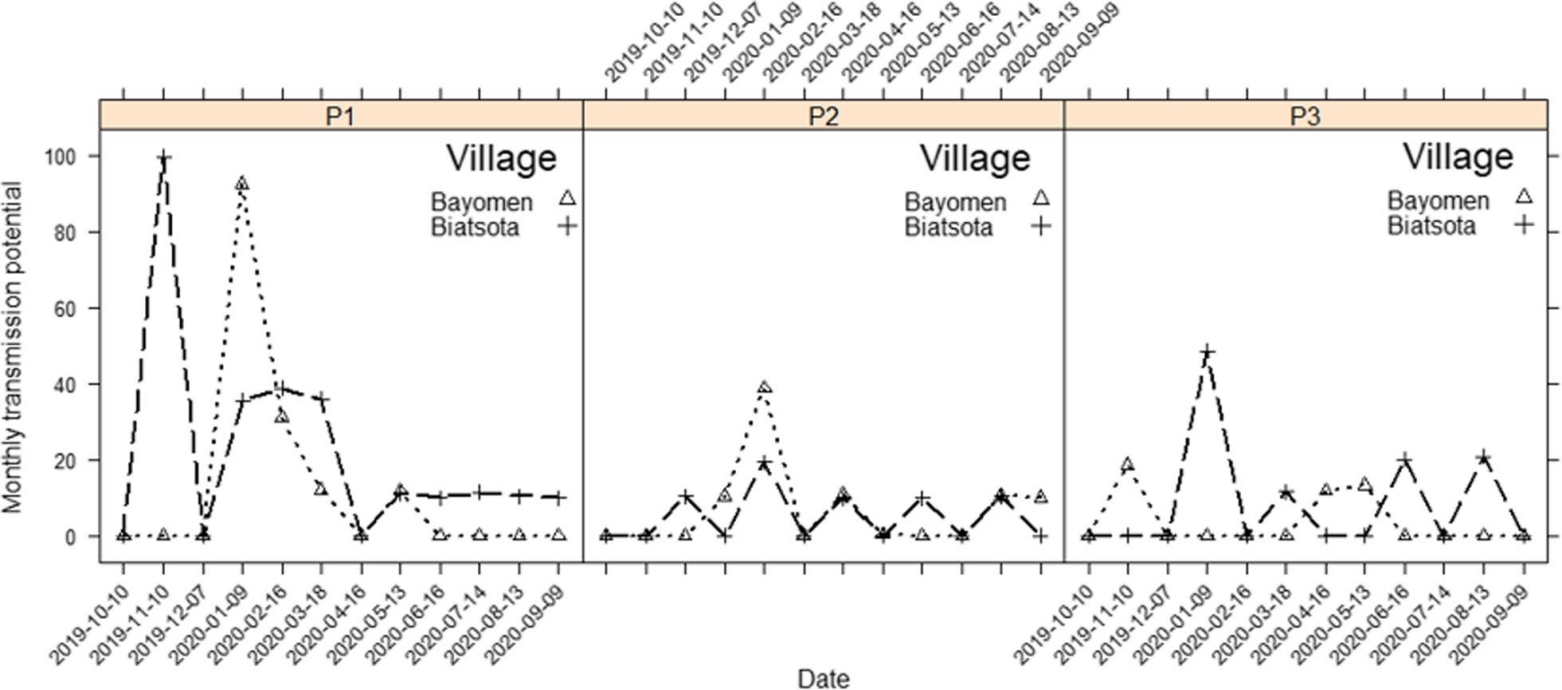
Monthly transmission potentials in each catch points in the study villages. For explanation of catch points P1, P2, P3, see Fig. 1 caption

Of the 57,517 dissected blackflies, a total of 100 harboured at least one filarial parasite morphologically indistinguishable from *O. volvulus* larvae (L1-L3), among which 54 were infective (L3 in the black fly mouth parts). Generally, MTPs at all catch points ranged between 0 and 92 infective larvae/human/year in Bayomen (with the peak in January) and between 0 and 100 infective larvae/human/month in Biatsota (with the peak in November) (Fig 4). The comparison of median MTPs between catch points of a village was found not to be significantly difference in Bayomen (*χ*^2^ = 0.635,* df* = 2, *P* = 0.728) and Biatsota (*χ*^2^ = 4.67,* df* = 2, *P* = 0.097). Likewise, no difference was found when comparing the MTPs between the two villages for catch points located at similar distances from riverbank (P1-P3) (*P* > 0.160). The ATPs were higher at the riverside in both villages: 165 infective larvae/human/year in Bayomen and 255 infective larvae/human/year in Biatsota (Table 1).

## Discussion

The objective of this study was to investigate the differences in MBR and ATP between three catch points to guide the choice of EWT placement for an optimal reduction of black fly population densities and onchocerciasis transmission in two first-line villages located in the Bafia Health District, Centre Region, Cameroon.

In general, the total number of black flies captured was similar in the two villages, indicating that the human populations living in these two villages suffer comparable nuisance from *O. volvulus* vectors. The biting rates also showed similar trends in the two villages, with the vector density decreasing with increasing distance from the riverside to the centre of the villages. Indeed, after their emergence, female black flies gradually migrate from breeding sites (in the rivers) towards the centre of the villages, searching for hosts on which they can feed. As a result, the level of exposure to these vectors varies considerably according to distance from the river. Although no significant difference was found between catch points located at the same distance from the river in the two villages, biting rates were generally higher in Bayomen than in Biatsota. This difference suggests that transmission rates may also be higher in Bayomen and, therefore, that more effort may be needed to stop transmission in this village compared to Biatsota. The highest bite rates were recorded in November, which marks the beginning of the long dry season in both villages. Indeed, by the end of the rainy season, the breeding sites are inundated with large volumes of water. With the onset of the dry season, these submerged breeding sites re-appear, providing sites for female black flies to lay their eggs and subsequently leading to the progressive increase in adult black fly, similarly to the cycles previously documented with tsetse flies [22] This observation suggests that there is a natural regulation of black fly populations that should be considered when planning vector control activities.

The proportion of parous black flies was higher on the riverbank than in the other catch points in the Biatsota community; to the contrary, in Bayomen, the highest proportion of parous black flies was found at the centre of the village. This result could be explained by the presence of the ferry at the Biatsota riverbank; black flies collected at that site emerged from breeding sites located not far from the ferry where human activities are concentrated and, therefore, there is a permanent availability of hosts on which the flies can feed. In contrast, human population density is a more important factor in the Bayomen village, and almost no human activities take place at the riverside, possibly explaining the higher PR observed towards the centre of the village. Parity rates are of particular importance when investigating *O. volvulus* transmission and disease epidemiology, as only flies that have already laid eggs at least once may harbour infective L3 larvae. Consequently, if parity rates in black fly populations are high, parasite transmission will be more likely to be high compared to populations with lower PRs, all other factors being equal. Based on this information, it could be hypothesised that transmission can be more effective on the riverbanks of the Mbam in Biatsota village where human activities take place throughout the day. This situation could explain why onchocerciasis is persisting in this village with a high prevalence despite more than two decades of mass ivermectin distribution [11].

The epidemiological importance of the observed vector density gradient was further supported by data from the dissection of black flies and evaluation of entomological indicators. Although there was no significant difference between MTPs at catch points within each village in this study, the highest ATPs were recorded at the riversides where blackflies are the most abundant, as was previously described in other studies conducted in Cameroon [23, 24]. This result was confirmed by pool screening, with the highest infection rates being recorded at the riverbanks in the two villages, possibly explained by the fact that most of the infected female black flies—that is, those that already had a blood meal—are found around the breeding sites where they will lay their eggs and are therefore captured after oviposition. This finding also suggests that people living or working in the immediate vicinity of these breeding sites are also more exposed to black fly bites and *O. volvulus* infection than those living or working further away. Pool screening detected and confirmed the presence of *O. volvulus* parasite species at each of the catch points. Even though black flies were not screened for *O. ochengi*, it was anticipated that this species will be in the minority in this area, as was previously demonstrated [15].

To optimise the effectiveness of EWT in reducing black fly densities and therefore accelerating the elimination of onchocerciasis, the shape of the trap as well as the addition of attractants have been the main parameters usually taken into account [9, 10]. To the best of our knowledge, this is the first study to investigate the potential location where traps should be deployed for an optimal impact on vector density and transmission potential. Both of these parameters were found to be higher at the riverside of the two villages, indicating that there should be a priority for setting up EWT or other trapping systems at riverside locations for optimal performance within the framework of onchocerciasis control/elimination.

### Limitations

The molecular analyses carried out as part of this study were not performed on the larvae collected during dissection. However, given that the pool screening results showed the presence of *O. volvulus* in black flies and the results of the study by Hendy et al. study [15], which showed a low proportion of pools containing *O. ochengui* (7/417 body pools), the dissection-based results in this study and the transmission potentials obtained are considered to be reliable.

## Conclusions

This study reveals that there was a vector density gradient observed based on the distance to the river, with the highest ABRs and ATPs recorded at the riverside sites. The placement of EWTs and/or other trapping systems can be optimised to reduce black fly densities and contribute to the interruption of the transmission of onchocerciasis. Transmission potentials and parity rates should be considered while optimising trap placement.

## Data Availability

All data generated or analysed during this study are included in this published article and its supplementary information files.

## Abbreviations

ABR: Annual biting rate
ATP: Annual transmission potential
CDTI: Community-directed treatment with ivermectin
EWT: Esperanza Window Trap
Fw: Forward
MBR: Monthly biting rate
MTP: Monthly transmission potential
OCP: Onchocerciasis control program in West African
PR: Parous rate
Rev: Reverse
